# Glycosylation of IgG Associates with Hypertension and Type 2 Diabetes Mellitus Comorbidity in the Chinese Muslim Ethnic Minorities and the Han Chinese

**DOI:** 10.3390/jpm11070614

**Published:** 2021-06-29

**Authors:** Xiaoni Meng, Manshu Song, Marija Vilaj, Jerko Štambuk, Mamatyusupu Dolikun, Jie Zhang, Di Liu, Hao Wang, Xiaoyu Zhang, Jinxia Zhang, Weijie Cao, Ana Momčilović, Irena Trbojević-Akmačić, Xingang Li, Deqiang Zheng, Lijuan Wu, Xiuhua Guo, Youxin Wang, Gordan Lauc, Wei Wang

**Affiliations:** 1 Beijing Key Laboratory of Clinical Epidemiology, School of Public Health, Capital Medical University, Beijing 100069, China; mengxiaoni385@163.com (X.M.); zhangjie@ccmu.edu.cn (J.Z.); liudiepistat@163.com (D.L.); hydzxy@126.com (X.Z.); zjxccmu@163.com (J.Z.); weijiecaochris@163.com (W.C.); deqiangzheng@163.com (D.Z.); wujuan811017@163.com (L.W.); guoxiuh@ccmu.edu.cn (X.G.); wangy@ccmu.edu.cn (Y.W.); wei.wang@ecu.edu.au (W.W.); 2 School of Medical and Health Sciences, Edith Cowan University, Perth, WA 6027, Australia; xingang.li@ecu.edu.au; 3 Genos Glycoscience Research Laboratory, 10000 Zagreb, Croatia; marevilaj@gmail.hr (M.V.); jstambuk@genos.hr (J.Š.); A.Momcilovic@lumc.nl (A.M.); iakmacic@genos.hr (I.T.-A.); glauc@pharma.hr (G.L.); 4 College of the Life Sciences and Technology, Xinjiang University, Urumqi 830046, China; duolikun530@126.com; 5 Department of Clinical Epidemiology and Evidence-Based Medicine, National Clinical Research Center for Digestive Disease, Beijing Friendship Hospital, Capital Medical University, Beijing 100050, China; howard.hao.wang@hotmail.com; 6 Centre for Precision Health, Edith Cowan University, Perth, WA 6027, Australia; 7 Faculty of Pharmacy and Biochemistry, University of Zagreb, 10000 Zagreb, Croatia

**Keywords:** hypertension and type 2 diabetes mellitus comorbidity, IgG, N-glycosylation, biomarkers

## Abstract

Objectives: Hypertension and type 2 diabetes mellitus comorbidity (HDC) is common, which confers a higher risk of cardiovascular disease than the presence of either condition alone. Describing the underlying glycomic changes of immunoglobulin G (IgG) that predispose individuals to HDC may help develop novel protective immune-targeted and anti-inflammatory therapies. Therefore, we investigated glycosylation changes of IgG associated with HDC. Methods: The IgG N-glycan profiles of 883 plasma samples from the three northwestern Chinese Muslim ethnic minorities and the Han Chinese were analyzed by ultra-performance liquid chromatography instrument. Results: We found that 12 and six IgG N-glycan traits showed significant associations with HDC in the Chinese Muslim ethnic minorities and the Han Chinese, respectively, after adjustment for potential confounders and false discovery rate. Adding the IgG N-glycan traits to the baseline models, the area under the receiver operating characteristic curves (AUCs) of the combined models differentiating HDC from hypertension (HTN), type 2 diabetes mellitus (T2DM), and healthy individuals were 0.717, 0.747, and 0.786 in the pooled samples of Chinese Muslim ethnic minorities, and 0.828, 0.689, and 0.901 in the Han Chinese, respectively, showing improved discriminating performance than both the baseline models and the glycan-based models. Conclusion: Altered IgG N-glycan profiles were shown to associate with HDC, suggesting the involvement of inflammatory processes of IgG glycosylation. The alterations of IgG N-glycome, illustrated here for the first time in HDC, demonstrate a biomarker potential, which may shed light on future studies investigating their potential for monitoring or preventing the progression from HTN or T2DM towards HDC.

## 1. Introduction

Hypertension (HTN) is one of the leading modifiable risk factors for morbidity and mortality of cardiovascular disease (CVD) worldwide [1]. Likewise, type 2 diabetes mellitus (T2DM) has become one of the global disease burdens [2]. HTN and T2DM are closely interlinked due to similar risk factors including obesity, dyslipidemia, insulin resistance, endothelial dysfunction, vascular inflammation, atherosclerosis, and sequential inappropriate activation of renin-angiotensin system, etc. [3,4]. Previous studies have shown that hypertension is at least twice as frequent in patients with diabetes compared with those who do not have diabetes [5]. Accordingly, a considerable portion of the world population undergoes coexistent T2DM and HTN, which increases the risk of CVD and renal failure in individuals [6]. Compared with the normotensive non-diabetic individuals, patients with HTN and T2DM comorbidity (HDC) have a four-fold increased risk of CVD [7]. The number of patients diagnosed with HTN and T2DM comorbidity (HDC) is on the rise, resulting in increased medical expenses and resource utilization [8]. Thus, further studies of biomarkers for monitoring and preventing the progression from HTN or T2DM towards HDC are timely and called for.

The glycan attachment to individual proteins is driven by specific enzymes and plays an indispensable role in almost all physiological processes [9]. In general, N-glycome constructed in healthy individuals is fairly stable but can vary due to pathological conditions, especially during inflammation [10]. Altered glycosylation pathways can have significant effects on protein structure and function that occur during the transition from healthy to diseased states. Due to the absence of the genetic template and the lack of stringency within the biosynthetic pathway, the glycan structures of individuals are influenced by the intricate interplay of genetic polymorphisms and environmental factors [11]. Therefore, it is warranted to elucidate the role of altered glycome composition in the pathogenic immune response and to analyze the possibility of using N-glycosylation as a latent predictor for the development of multifactorial diseases, such as HDC.

As the most abundant antibody in human blood, immunoglobulin G (IgG) regulates systemic inflammation balance at multiple levels, and its function is largely influenced by glycosylation. It has been shown that the pro- and anti-inflammatory activities of IgG could no longer be demonstrated if N-glycans are completely removed [12]. Aberrant IgG N-glycosylation has also been demonstrated to be involved in the pathogenesis such as aging [13], HTN [14], and T2DM [15,16], which are relevant risk factors contributing to HDC. These observations make us speculate that changes of IgG N-glycosylation may be related to the pathogenesis of HDC by regulating inflammation. Understanding the underlying altered IgG N-glycosylation profiles that predispose individuals to HDC may help to develop novel protective immune-targeted and anti-inflammatory therapies and/or prevention approaches. However, the associations between IgG N-glycans and HDC have not been described in current literature.

There are 55 officially recognized ethnic minority groups within China in addition to Han majority (which constitute about 91.9% of the total population). The population size of Han Chinese and Chinese ethnic minorities are 1286.3 and 125.5 million, respectively (according to the seventh national census statistics of China conducted in 2020). The genetic background, culture, socioeconomic conditions, climate, geographical features, lifestyle, and dietary habit are substantially different between Han Chinese and other ethnic minorities. The Kirgiz (KGZ), Tajik (TJK), and Uygur (UIG) are three Chinese Muslim ethnic minorities, with a total population size of 11.94 million, inhabiting the Xinjiang Uygur Autonomous Region, the northwestern frontier of China, for generations, without intermarry with other ethnic groups [17]. These Chinese Muslim minorities share relatively similar lifestyles, diet-related habits, living environment, culture, and genetic backgrounds, thereby are well-defined isolated populations with substantial ancestry contribution from Western Eurasian people [18,19]. The prevalence of HTN and T2DM in the northwestern Chinese minority populations is on the rise and relatively higher than that in the Han Chinese, the majority ethnic group in China [20,21]. Investigating the IgG N-glycan profiles of ethnic groups with different prevalence of HTN and T2DM could shed light on the potential population-specific and/or general IgG N-glycan patterns and their relevance to the pathogenesis of HDC.

In this study, we investigated the changes in IgG N-glycosylation profiles associated with HDC and their biomarker potential for distinguishing HDC from HTN, T2DM, and healthy individuals in the aforementioned populations. This work could further increase our understanding of HDC pathophysiology and may shed light on future studies investigating their potential for monitoring or preventing the progression from HTN or T2DM towards HDC.

## 2. Materials and Methods

### 2.1. Subjects

The participants from the three northwestern Chinese ethnic minorities (KGZ, TJK, and UIG) were recruited, and their demographic characteristics (gender, age, ethnicity, levels of education, occupation, and urban/rural residence) and anthropometric measurements (height, weight, body mass index [BMI], and blood pressure), as well as blood samples, were collected. All the Muslim participants were recruited from the isolated underdeveloped rural areas (Halajun town, Tagan town, and Minfeng town) of Xinjiang, China, where the respective Muslim minority “exclusively” inhabit for generations without co-resident Han or other ethnic groups, with either primary or secondary education. The occupations of all the UIG participants were farmers and of all the KGZ and TJK participants were herdsmen. A total of 484 samples from 165 KGZ, 146 TJK, and 173 UIG were included in this study, composed of 67 HDC, 183 HTN, 51 T2DM, and 183 healthy individuals (median ages for the four health condition groups: 63, 59, 54, and 51 years). The participants met the following inclusion criteria: (1) Over 18 years old; (2) self-reported the KGZ, TJK, or UIG ethnicity without intermarriage history with other ethnic groups within at least the past three generations. Individuals were excluded based on the following criteria: (1) Pregnant or lactating women; (2) history of severe hepatic, nephritic and autoimmune disease; and (3) diagnosis of diseases of mental illness or infectious disease.

In this study, a total of 399 Han Chinese participants from a community-based survey in Beijing were also included, composed of 72 HDC, 112 HTN, 50 T2DM, and 165 healthy controls (median ages for the four health condition groups: 66, 49, 54, and 46 years). The demographic characteristics, anthropometric measurements, and blood samples of Han Chinese population were collected in a cross-sectional study conducted in 2012, as described previously [13]. 

All 883 participants included in this study have signed written informed consent. The study was approved by the local community leaders and the Ethics Committee of Capital Medical University, Beijing, China (No. 2015SY21 and 2009SY16) and adhered to the principles of the Declaration of Helsinki.

### 2.2. Diagnostic Criteria

HTN was defined as an average systolic blood pressure (SBP) ≥ 140 mmHg and/or an average diastolic blood pressure (DBP) ≥ 90 mmHg, according to the Chinese guidelines for the prevention and treatment of HTN in adults [22]. Peripheral blood samples of the participants for analyses of biochemical indicators (fasting plasma glucose and blood lipids) and IgG N-glycan profiles were collected using EDTA anti-coagulated tube after an overnight fast. The diagnosis of T2DM was based on fasting blood glucose ≥ 7.0 mmol/L in this study, according to World Health Organization (WHO) Criteria [23]. HDC was defined as the co-existence of HTN and T2DM [24]. Dyslipidemia was defined as total cholesterol (TC) ≥ 6.2 mmol/L, triglycerides (TG) ≥ 2.3 mmol/L, low-density lipoprotein cholesterol (LDL) ≥ 4.1 mmol/L, or high-density lipoprotein cholesterol (HDL) < 1.0 mmol/L, according to the Guidelines for Prevention and Treatment of Dyslipidemia in Adults in China [25].

### 2.3. Analysis of IgG N-glycosylation

IgG was isolated from plasma samples from all participants using 96-well protein G monolithic plates, and N-linked glycans were released and labeled as described previously [26]. In brief, the isolated IgG was eluted from protein G with 1 mL of 0.1 M formic acid and instantly neutralized with 1 M ammonium bicarbonate. Then, IgG N-glycans were released using PNGase F, labeled with 2-aminobenzamide and purified with 96% acetonitrile and ultrapure water to remove proteins, salts and excess of reagents from deglycosylation and fluorescent labeling reaction. In the end, IgG N-glycans were analyzed by hydrophilic interaction liquid chromatography (HILIC) using ultra-performance liquid chromatography (UPLC) instrument as described previously [27].

In total, 24 chromatographic peaks, (i.e., IgG glycan peaks (GPs, GP1–GP24)) were measured and the amount of glycans in each peak was expressed as percentage of total integrated area [15], and the detailed structures of the N-glycan peaks were shown in Table S1. The 54 derived glycan traits (DGs, DG1–DG54) were calculated from the directly measured GPs [28]. These DGs were used to describe the relative abundance of galactosylation, sialylation, bisecting *N*-acetylglucosamine (GlcNAc), and core fucosylation. To remove the experimental variation from measurements, normalization and batch correction were conducted on the UPLC measured glycan data [29]. Therefore, the measurements were comparable across all samples.

### 2.4. Statistical Analysis

Based on our previous publications, in which the correlation coefficients of the relationship between the sialylated glycans (i.e., IgG 4 G1S (that is, Galactose and Sialic acid) and monosialylated glycan, respectively) and SBP in the Chinese Muslim ethnic minority (Kazakh) and the Han Chinese were of a similar magnitude: −0.373 and −0.372, respectively [30,31]. With 75% power and a 0.05 two-sided significance level, the required sample size is estimated to be 48 participants for each of the four health condition groups (i.e., HDC, HTN, T2DM, and healthy individuals) using StatsToDo (http://www.statstodo.com/SSizCorr_Pgm.php; accessed on 9 October 2020). The data from the three Chinese Muslim ethnic minorities with similar genetic and environmental backgrounds were pooled together for the statistical analysis, given that the sample size of the respective four health condition groups of the individual Chinese Muslim ethnic minority is insufficient.

Normality distributions of all glycan traits were double-checked by using the Kolmogorov–Smirnov test and Shapiro–Wilk test. When the continuous variable conformed to the normality distribution, means together with standard deviations were used in descriptive statistics. Otherwise, medians together with the interquartile ranges were used. The Chi-square test was applied to investigate the differences in categorical variables. Since the majority of the IgG N-glycan traits were not normally distributed, the Kruskal–Wallis test was conducted to determine the differences of directly measured and derived IgG N-glycan traits among the HDC, HTN, T2DM, and healthy controls. 

The IgG glycosylation features of HDC may combine the IgG glycosylation features of both HTN and T2DM, thus a Venn diagram was constructed to illustrate shared IgG N-glycan features between the four health condition groups using online software (http://bioinformatics.psb.ugent.be/webtools/Venn/; accessed on 17 April 2021). The associations between HDC and IgG N-glycan traits were assessed through logistic regression analysis, with the IgG N-glycan traits as independent variables, adjusted for potential confounders (i.e., age, gender, ethnicity, BMI, and dyslipidemia). Given the potential internal associations among IgG N-glycan traits, which could induce multicollinearity in the statistical models, the least absolute shrinkage and selection operator (LASSO) method was conducted to reduce dimension before constructing the logistic regression models.

To evaluate differences in IgG N-glycan traits of different health conditions in the three Chinese Muslim minorities pooled samples and Han Chinese samples, the IgG N-glycan traits screened by the LASSO method were included to construct logistic regression models for HDC versus HTN, HDC versus T2DM, and HDC versus healthy controls, respectively. Three types of logistic classification models were applied: (1) The baseline models included age, gender, ethnicity, BMI, and dyslipidemia as the covariates; (2) the glycan-based models included the screened IgG N-glycan traits as the covariates; and (3) the combined models incorporated the screened IgG N-glycan traits on top of the covariates included in the baseline models. The prediction error of the five-fold cross-validation procedure was used to assess the performance of the discriminant models (R package “*boot*”) [32]. The discriminant ability of the models in delineating the HDC from the HTN, T2DM, and healthy individuals was evaluated by receiver operating characteristic (ROC) curve analysis (R package “*pROC*”).

Data were analyzed and visualized using SAS (Version 9.4) and R packages (Version 4.0.4). For the logistic regression analysis, the Benjamini–Hochberg procedure was used to control the false discovery rate (FDR) [33]. All analyses were two-sided, and statistical significance was set at *p* < 0.05. Bonferroni adjustment was used for multiple comparisons among the four health condition groups to control familywise error rate (FWER) in a very stringent criterion and to compute the adjusted *p* values by directly adjust the significance level as *α*′ = *α*/*m* (i.e., the number of simultaneously tested hypotheses). Thus, *p* < 0.05/6 (0.0083) was considered to be statistical significance in the comparative analysis of demographic and biochemical characteristics, and the levels of IgG N-glycans between the groups.

## 3. Results

### 3.1. Demographic and Biochemical Characteristics

A total of 883 participants (484 Chinese Muslim minorities and 399 Han Chinese) were analyzed in this study. Demographic and biochemical characteristics of the HDC, HTN, T2DM, and healthy individuals for the Chinese Muslim ethnic minorities and those for the Han Chinese are shown separately (Table S2) and pooled together in Table 1. In the pooling of the Chinese Muslim ethnic minorities, the age significantly differed in HDC compared to T2DM and healthy controls, and the SBP and DBP were significantly higher in the HDC group than those in both T2DM and healthy individuals, and the FBG was higher in the HDC than that in both the HTN and healthy individuals. In the Han Chinese individuals, the age, gender, BMI, SBP, DBP, TC, TG, LDL, FBG, and dyslipidemia significantly differed among the four health condition groups (i.e., HDC, HTN, T2DM, and healthy controls).

### 3.2. The Association of IgG N-Glycans with HDC

**Figure 1:**
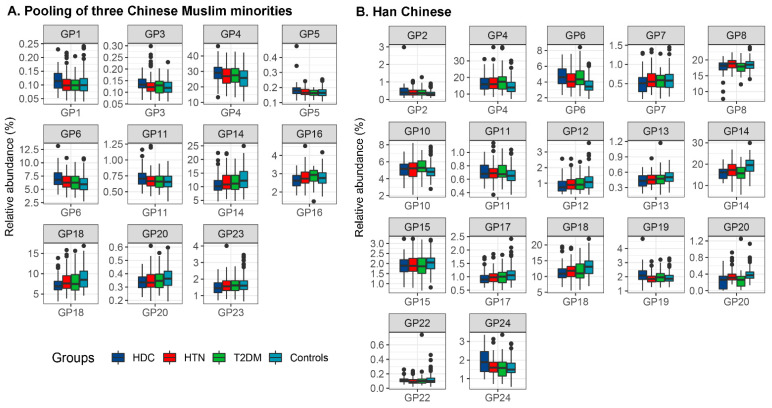
Differences in IgG N-glycan traits between HDC, HTN, T2DM patients, and healthy controls for the pooled samples from three Chinese Muslim ethnic minorities (**A**) and the Han Chinese samples (**B**). Each box represents the 25th to 75th percentiles (interquartile range [IQR]). Lines inside the boxes represent the medians. The whiskers represent the lowest and highest values within the boxes ±1.5 the IQR. GP, glycan peak; HDC, hypertension and type 2 diabetes mellitus comorbidity; HTN, hypertension; T2DM, type 2 diabetes mellitus.

IgG N-glycome composition (i.e., GPs and DGs) was analyzed in all samples. Out of the total 78 glycan traits, 56 and 62 glycan traits were found to be abnormally distributed (*p*_Kolmogorov–Smirnov_ < 0.05 and *p*_Shapiro–Wilk_ < 0.05) in the pooled samples from the three Chinese Muslim ethnic minorities and those from Han Chinese, respectively (Table S3). The differences of directly measured and derived glycan compositions among HDC, HTN, T2DM, and healthy controls for the Chinese Muslim ethnic minorities and the Han Chinese, respectively, were shown in Figure 1 and Table S3. We found the increased relative abundance of GP4 (FA2), GP6 (FA2B), and GP11 (FA2[3]BG1) and the reduced relative abundance of GP14 (FA2G2), GP18 (FA2G2S1), and GP20 (FA2FG2S1) in the HDC group compared with the other three health condition groups (i.e., HTN, T2DM, and healthy individuals), with similar trends in the Chinese Muslim ethnic minorities and the Han Chinese (Figure 1).

**Figure 2:**
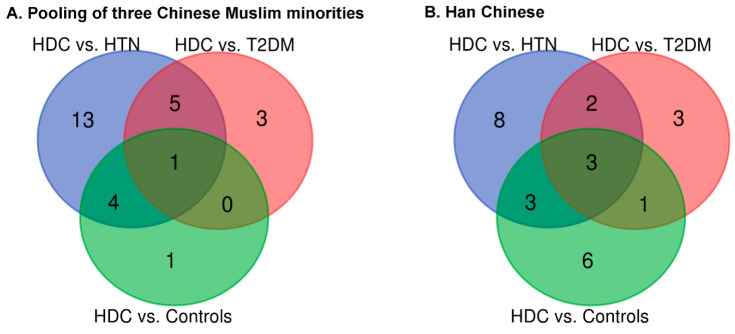
Venn diagram of shared and unique IgG N-glycan features between the different health condition groups for the pooled samples from three northwestern Chinese Muslim ethnic minorities and those from the Han Chinese. The numbers of shared and unique IgG N-glycan traits in the three pairwise comparison groups are shown based on the result of logistic regression analysis adjusted for the covariates (for the pooling of three Chinese Muslim minorities, logistic regression adjusted for age, gender, ethnicity, BMI, and dyslipidemia [A]; For the Han Chinese, logistic regression adjusted for age, gender, BMI, and dyslipidemia [B]). HDC, hypertension and type 2 diabetes mellitus comorbidity; HTN, hypertension; T2DM, type 2 diabetes mellitus.

**Figure 3:**
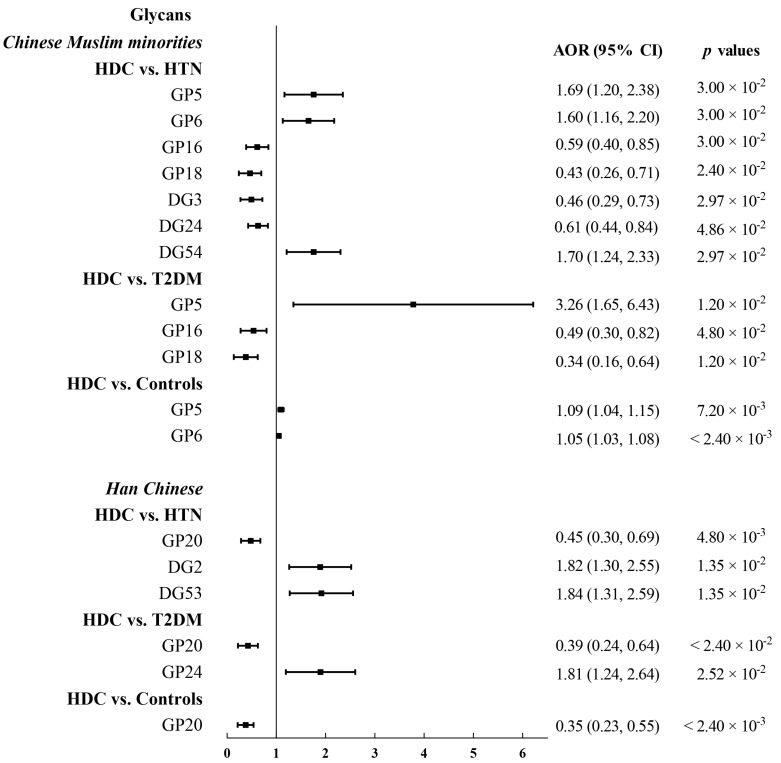
The associations between IgG N-glycan traits and HDC. AORs and 95% Cis for the association of IgG N-glycan traits with HDC versus HTN/T2DM/healthy controls adjusted for the covariates and false discovery rate (for the pooling of three Chinese Muslim minorities, logistic regression adjusted for age, gender, ethnicity, BMI, and dyslipidemia; For the Han Chinese, logistic regression adjusted for age, gender, BMI, and dyslipidemia). FDR was controlled by Benjamini–Hochberg method and *p* < 0.05 was considered statistical significance. AOR, adjusted odds ratio; BMI, body mass index; CI, confidence interval; DG, derived glycan; FDR, false discovery rate; GP, glycan peak; HDC, hypertension and type 2 diabetes mellitus comorbidity; HTN, hypertension; T2DM, type 2 diabetes mellitus.

Table S4 and its accompanying Venn Diagrams (Figure 2) displays and illustrates respectively, the results of the logistic regression analysis of IgG N-glycans after adjustment for potential confounders (i.e., age, gender, ethnicity, BMI, and dyslipidemia), and shared and unique IgG glycan features between the different health conditions. Noteworthily, 12 (i.e., 7 in HDC versus HTN, 3 in HDC versus T2DM, and 2 in HDC versus Controls) and 6 (i.e., 3 in HDC versus HTN, 2 in HDC versus T2DM, and 1 in HDC versus Controls) IgG N-glycan traits were found to be significantly different between four health condition groups within the three Chinese Muslim minorities pooled samples and Han Chinese samples, respectively, after controlling the aforementioned confounders and FDR, in the HDC pairwise comparison with the three health condition groups (i.e., HDC versus HTN, HDC versus T2DM, and HDC versus healthy individuals) (Table S4 and Figure 3). To elaborate further, it was found that within the pooled samples from the three Chinese Muslim minorities, seven glycan traits (increased relative abundance of GP5 [M5], GP6 [FA2B], and DG54 [BG2^n^/(FG2^n^ + FBG2^n^)] as well as reduced relative abundance of GP16 [FA2G1S1], GP18 [FA2G2S1], DG3 [FGS/(F + FG + FGS)], and DG24 [GP8^n^]) were significant differences between HDC and HTN (*p* < 0.05). Three glycan traits (increased relative abundance of GP5 and reduced relative abundance of GP16 and GP18) were significant differences between HDC and T2DM (*p* < 0.05). In addition, two glycan traits (GP5 and GP6), with increased relative abundance, were significantly different between HDC and healthy controls (*p* < 0.05).

For the pooling of three Chinese Muslim ethnic minorities, we found that one shared glycan trait—GP5, containing high mannose glycan (M5) in the HDC, was significantly higher than those in the other three healthy condition groups (i.e., HTN, T2DM, and healthy individuals). Additionally, two shared glycan traits (GP16 and GP18), containing core-fucosylated galactosylated glycans with sialic acid (FA2G1S1 and FA2G2S1, respectively), were observed in lower levels in the HDC compared with HTN and T2DM, and a core-fucosylated agalactosylated glycan containing bisecting GlcNAc eluting in GP6 (FA2B) was observed in higher levels in the HDC than those in the HTN and healthy individuals (Figure 3 and Table S4).

Similarly, for the Han Chinese, in comparisons between HDC and the other three healthy condition groups (i.e., HTN, T2DM, and healthy controls), one shared glycan trait (GP20 [FA2FG2S1]) containing core-fucosylated digalactosylated glycan with sialic acid of reduced relative abundance was of statistical significance. The relative abundance of DG2 (FBGS/(FBG + FBGS)) and DG53 (FG2^n^/(BG2^n^ + FBG2^n^)) in the HDC were significantly higher than those in the HTN. Additionally, the increased relative abundance of GP24 (FA2BG2S2) containing sialylation of fucosylated galactosylated structures with bisecting GlcNAc was found in the HDC compared with the T2DM (Figure 3 and Table S4).

### 3.3. The Comparison of IgG N-Glycans between the Chinese Muslim Ethnic Minorities Pooled Samples and the Han Chinese Samples

We observed the increased relative abundance of sialylated glycans (i.e., GP24, DG2, and DG53) in the Han Chinese with HDC (HDC versus HTN or T2DM), while a reduced trend (i.e., GP16, GP18, and DG3) was presented in the Chinese Muslim ethnic minorities pooled samples with HDC (HDC versus HTN or T2DM). The HDC-associated changes in the sialylated glycans showed different trends between the Chinese Muslim ethnic minorities and the Han Chinese as a consequence of the presence or absence of bisecting GlcNAc in these sialylated glycans in the different ethnic groups.

Although the logistic regression analyses suggested that directly measured and derived IgG N-glycans between the three Chinese Muslim ethnic minorities and the Han Chinese in the three pairwise comparison groups (i.e., HDC versus HTN, HDC versus T2DM, and HDC versus healthy controls) did not share similarities in their results, we found that their respective results all presented low levels of core-fucosylation and galactosylation (i.e., GP16, GP18, and GP20), as well as high levels of bisecting GlcNAc (i.e., GP6, DG54, GP24, and DG53) in the three Chinese Muslim ethnic minorities with HDC and also in the Han Chinese with HDC (Figure 3 and Table S4).

### 3.4. Development of the Classification Models and Discrimination of HDC

**Figure 4:**
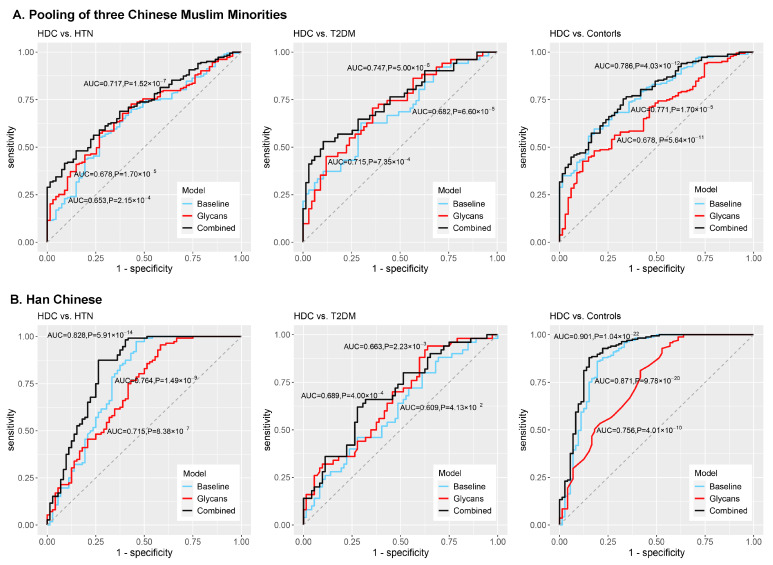
ROC curve illustrated the performance of the models in the classification of HDC from HTN, T2DM, and healthy individuals for the pooled samples from three Chinese northwestern Muslim ethnic minorities (**A**) and those from Han Chinese (**B**). When glycan traits are added into the combined models, the AUCs indicated that the IgG glycan profiles provide more information reinforcing the baseline models utilizing gender, age, ethnicity, BMI, and dyslipidemia as the covariates (with difference between 2 AUCs (95% CI) of 0.064 (0.002, 0.126), 0.065 (−0.009, 0.139), and 0.015 (−0.011, 0.042) in distinguishing HDC from HTN, T2DM, and healthy controls, respectively, in the pooling of three Chinese Muslim ethnic minorities, and of 0.064 (0.008, 0.118), 0.080 (−0.013, 0.172), and 0.030 (0.003, 0.055), respectively, in the Han Chinese). AUC, area under the receiver operating characteristic curve; CI, confidence interval; HDC, hypertension and type 2 diabetes mellitus comorbidity; HTN, hypertension; T2DM, type 2 diabetes mellitus; ROC, receiver operating characteristic curve.

Three types of logistic classification models (i.e., baseline models, glycan-based models, and combined models) were constructed for distinguishing HDC from HTN, T2DM, and healthy individuals, and were trained and evaluated by the five-fold cross-validation procedure and ROC curve analysis (Figure 4 and Table 2). The detailed information on the covariates included in the logistic classification models was shown in Table 2. Specifically, in the pooling of three Chinese Muslim ethnic minorities, the AUCs of the glycan-based models discriminating HDC from HTN and T2DM were 0.678 and 0.715, respectively, showing limited improved discriminative performance than the baseline models. However, the AUC of the glycan-based model discriminating HDC from healthy controls was lower than that of the baseline model (difference between AUCs, 0.093, *p* = 3.97 × 10^−2^). A similar trend was observed in the Han Chinese for discriminating HDC from healthy controls (difference between AUCs, 0.115, *p* = 9.56 × 10^−3^) (Table S5).

Adding both IgG N-glycan and baseline traits to the combined models, the AUCs of the combined models differentiating HDC from HTN, T2DM, and healthy controls were 0.717, 0.747, and 0.786 in the pooling of three Chinese Muslim ethnic minorities, and 0.828, 0.689, and 0.901 in the Han Chinese, respectively (Table 2). The AUCs showed modest improvement in the combined models compared to the baseline models, with increments of 0.064 (*p* = 4.18 × 10^−2^) for HDC versus HTN, 0.065 (*p* = 8.66 × 10^−2^) for HDC versus T2DM, and 0.015 (*p* = 2.45 × 10^−1^) for HDC versus healthy controls, in the pooling of three Chinese Muslim ethnic minorities; and with increments of 0.064 (*p* = 2.22 × 10^−2^) for HDC vs. HTN, 0.080 (*p* = 8.99 × 10^−2^) for HDC vs. T2DM, and 0.030 (*p* = 2.73 × 10^−^^1^) for HDC vs. healthy controls, in the Han Chinese (Table S5). The discriminative performances of the combined models were shown to be superior to the baseline models. Although not all the AUCs of the combined models are statistically different from the baseline models, the addition of glycan variables into the baseline models can increase the discriminative power of the models. It suggests that IgG N-glycans could reinforce the discriminative potential of models. Additionally, all but the combined model discriminating HDC from HTN (Chinese Muslim ethnic minorities) showed lower prediction error than the baseline and glycan-based models in five-fold cross-validation (Table 2).

## 4. Discussion

To the best of our knowledge, this is the first investigation on the association and discriminative potential of IgG N-glycan traits in HDC versus HTN, T2DM, and healthy individuals in the pooling of three Chinese Muslim ethnic minorities (KGZ, TJK, and UIG), inhabiting the northwestern frontier of China, as well as the Han Chinese population.

HTN and T2DM are common comorbidities that are multifactorial diseases driven by many common pathogenic factors, including modifiable risk factors (obesity, dyslipidemia, insulin resistance, endothelial dysfunction, vascular inflammation, atherosclerosis, and sequential inappropriate activation of renin-angiotensin system, etc.) [3,4], environmental factors, and genetic predisposition [4,34]. IgG contains complex-type biantennary N-glycan structures, which activate or inhibit IgG Fcγ receptors (FcγRs) by altering the binding affinity of IgG, thereby decisively affecting effector functions of IgG [35]. The pro- and anti-inflammatory actions of IgG are modulated by the specific N-glycan residues including galactose, fucose, sialic acid, and bisecting GlcNAc [9,36,37]. Cumulative evidence indicates that the alteration of terminal modification in IgG N-glycan plays a vital role in the anti- or pro-inflammatory process. Previous studies reported that aberrant glycosylation of IgG (low levels of sialylation and core fucosylation, as well as the high level of bisecting GlcNAc) have been demonstrated to be involved in a range of specific health conditions related to HDC, including aging, obesity, HTN, T2DM, dyslipidemia, and metabolic syndrome [13,14,31,32,38,39,40]. In the present study, the altered IgG N-glycosylation profile was also observed in the HDC patients, further suggesting that IgG N-glycome patterns are associated with the progression of inflammatory disorders, such as HDC, via inflammatory signaling. These findings present a situation where the role of IgG glycans in the pathogenesis of HDC extends beyond just a reflection of changes stemming from the onset of disease, but rather perhaps has some contribution towards influencing pathogenesis itself resulting from the altered inflammatory actions of IgG.

In this study, the higher level of GP5 (M5) and GP6 (FA2B), and the lower levels of GP16 (FA2G1S1) and GP18 (FA2G2S1) were found to be the population-specific IgG N-glycomic changes with statistical significance (*p* < 0.05) for the pooled samples from three Chinese Muslim ethnic minorities with HDC; and the lower level of GP20 (FA2FG2S1) were observed to be unique to the Han Chinese with HDC. In the pooled samples from the three Chinese Muslim ethnic minorities, HDC patients presented increased core-fucosylation with bisecting GlcNAc of IgG (GP6 and DG54), but decreased sialylation (GP16, GP18, and DG3) and monogalactosylated (GP16) and digalactosylated (GP18) core-fucosylated glycans without bisecting GlcNAc of IgG, when compared with HTN patients. In comparison with T2DM patients, HDC patients showed decreased galactosylation, sialylation, and core-fucosylation without bisecting GlcNAc of IgG (GP16 and GP18), but increased mannose structures of IgG (GP5). Additionally, compared to healthy individuals, HDC patients presented increased core-fucosylation with bisecting GlcNAc (GP6) and mannose structures of IgG (GP5). In the Han Chinese, the lower level of core-fucosylated galactosylated glycan containing sialic acid of IgG (GP20) was observed in the HDC compared with HTN/T2DM/healthy individuals, while sialylated glycans (reduced relative abundance of GP20 and increased relative abundance of GP24 [FA2BG2S2], DG2 [FBGS/(FBG + FBGS)], and DG53 [FG2^n^/(BG2^n^ + FBG2^n^)]) presented inconsistent trends in HDC patients. Specifically, the common characteristic of these HDC-associated sialylated glycans is the presence of bisecting N-GlcNAc, and the increase of this structural feature in HDC outweighed the effects of a decrease in sialylation, which is in agreement with the alterations of sialyation observed in the study on systemic lupus erythematosus [29].

Fucosylation plays key roles in the function of IgG and highly fucosylated IgG shows a reduced magnitude of antibody-dependent cellular cytotoxicity (ADCC), which is a result of IgG-subclass IgG1 binding to each of the different Fc receptors [41]. Bisecting GlcNAc is the central branch of N-glycans, which is biosynthesized by the glycosyltransferase GnT-III encoded by the MGAT3 gene [42]. The presence of bisecting GlcNAc in N-glycans could suppress various terminal modifications (fucosylation, sialylation, and others) of N-glycans [42], affecting the structure of glycans exerting noteworthy effects on inflammatory responses or immune function. IgG carrying glycans with bisecting GlcNAc was reported to have pronouncedly increased affinity to FcγRIIIa leading to an enhancement in ADCC, whereas fucosylation without bisecting GlcNAc of IgG can reduce ADCC [37]. Previous studies showed that such increased fucosylation with bisecting GlcNAc was associated with T2DM [15]. We also observed similarly increased fucosylation in structures with bisecting GlcNAc (GP6), and decreased fucosylation in structures without bisecting GlcNAc (GP16, GP18, and GP20) in the HDC patients, compared with HTN and T2DM patients. The increased fucosylation in structures with bisecting GlcNAc of IgG N-glycans could lead to the pro-inflammatory status of the body [37], thereby contributing to the progression of HTN or T2DM towards HDC. Thus, the present results indicate that the individuals with higher levels of fucosylated structures with bisecting GlcNAc of IgG may increase the risk for HDC, and that pathophysiological states of individuals with HDC are conditioned on a chronic inflammatory process.

The level of galactosylation has an essential impact on the inflammatory activities of IgG; lack of galactosylation was shown to associate with the pro-inflammatory autoimmune responses through activation of the complement cascade [43]. The down-regulation of galactosyltransferase activity in plasma cells and the activation of complement cascades were identified as potential mechanisms for the changes in IgG galactosylation [43]. As a modulator of inflammatory activity, high galactosylation of IgG Fc N-glycan promotes the association of the inhibitory receptor FcγRIIb and the lectin-like receptor dectin-1, which blocks the pro-inflammatory effector functions of C5aR and CXCR2, and enhances the anti-inflammatory activity of antigen-specific IgG1 immune complexes [44]. Previous studies have suggested that the decrease in galactosylation of IgG N-glycans play potential roles in the development and progression of chronic pro-inflammatory diseases, including HTN, T2DM, and dementia [14,15,16,45], which are in agreement with the alterations of IgG N-glycosylation patterns observed in the HDC patients in the present study. The decreased IgG galactosylation found in HDC presented a similar pattern found in the previous studies and thus our finding further supports the hypothesis that a decrease in galactosylation is a general feature of multiple chronic diseases associated with decreased anti-inflammatory/immunosuppressive potential of circulating IgG.

Besides fucosylation and galactosylation, sialylation mediates partly the inflammatory function of IgG. IgG shows different pro- and anti-inflammatory potentials on the basis of different Fcγ receptors that IgG preferentially binds to, and the affinity of the receptor depends on the composition of the glycans attached to the 297 asparagine [46]. The addition of sialic acid to IgG can decrease its affinity for FcγRIII, thereby reducing the degree of ADCC, converting its function from pro- to anti-inflammatory [47]. In the two comparison groups (HDC versus HTN, HDC versus T2DM), we observed that patients with HDC showed a significant decrease in sialylation (GP16, GP18, and GP20), indicating that sialylation is correlated with HDC through decreasing adaptive pro-inflammatory actions of IgG. However, sialylation with bisecting GlcNAc showed the reduced relative abundance in HDC patients, which was consistent with the suppression of core fucosylation expression due to the presence of bisecting GlcNAc. Bisecting GlcNAc inhibits the biosynthesis and modification of sialic acid of terminal epitopes in N-glycans, which results in the relative abundance of sialylation (GP16, GP18, GP20, GP24, DG2, DG3, and DG53) presenting inconsistent trends in HDC patients. Additionally, we found the decrease of GPs containing galactosylated sialylated glycan structures (GP16, GP18, and GP20) in HDC patients. Since terminal sialic acid residues are covalently attached to galactosylated glycans, the decrease of galactosylated sialylation could be a consequence of a decrease in galactosylation [48], which could provide a plausible explanation for the phenomenon of the co-directional changes of sialylation and galactosylation shown in the HDC patients. It is worthwhile to investigate the potential of the level of sialylation and galactosylation of IgG for monitoring the progression from HTN or T2DM towards HDC. Thus, future prospective cohort studies are called for to explore if there is a stepwise decrease trend in levels of sialylation and galactosylation of IgG during progression from healthy individuals to HTN or T2DM and then to HDC.

Among the different Chinese ethnic minorities, the similarities and differences in the glycosylation patterns were found in both the HTN and T2DM patients, compared with the HDC patients, suggesting that those individuals who have developed T2DM or HTN do not show consistent alterations of IgG N-glycans. More work is required on understanding alterations of IgG N-glycans in HTN or T2DM individuals to see if their alterations over time show an inclination towards HDC. Such IgG N-glycans profiling will help to contribute towards developing potential glycan biosignatures for monitoring the progression from HTN or T2DM towards HDC.

The AUCs of the combined models, based on the glycan traits and the baseline characteristics, demonstrate higher discriminative performance to help discern between HDC, HTN, T2DM, and healthy individuals than the models based on either the glycan traits or the baseline characteristics alone. IgG N-glycan profiles reflect the integrative effects of genetic, metabolic, and environmental factors, contributing significantly towards their discriminative potential [11]. Although the addition of glycan traits to the baseline models did not substantially improve the discrimination in all the combined models, the glycan based models constructed based on IgG N-glycans alone displayed enough discrimination power and all the combined models showed improved discrimination performance. These results suggest that IgG N-glycan profiles may contribute significantly to much of the combined risk of these characteristics, where dynamic and population-specific IgG N-glycomic changes can serve as functional effectors of the disease potentially increasing the diagnostic accuracy for complex diseases. The IgG N-glycosylation profiles associated with HDC described in this study may thus help to shed light on future studies investigating their biomarker potential for personalized monitoring and prevention of the progression from HTN or T2DM towards HDC.

The strength of this study is that we evaluated IgG N-glycomes in relation to HDC and explored similarities and differences in the IgG N-glycosylation profiles for the first time, particularly in different Chinese ethnic groups, including the Chinese Muslim minorities. The alterations of IgG N-glycosylation in HDC patients found in this study suggest a theoretical foundation and present practical direction for future studies to further elaborate the association of IgG glycosylation with HDC. These suggestions are, however, built upon the constraints of this study. Firstly, due to the small sample size of HDC in each ethnic minority in the present study, it is unfeasible to analyze the association of IgG N-glycans with HDC in each of the Muslim minorities separately. Consequently, the same health condition groups from the three Chinese Muslim ethnic minorities were pooled together for the analysis. Furthermore, the Han subjects included in the study were not co-resident in the Xinjiang Autonomous Region since the Muslim minorities were recruited from the isolated rural areas in Xinjiang, where the respective Muslim minority “exclusively” inhabit for generations without co-resident Han or other ethnic groups. Additionally, it is difficult to match the Han subjects with the Muslim minorities in terms of their profile of demographic variables due to the relatively small sample size of the respective health condition group of the Muslim minorities. To compensate for the potential impact of unmatched demographic variables on the results, we adjusted the potential confounding factors in the logistic regression analyses. Thirdly, the case-control nature of the present study was not able to provide the information about a causal relationship between the altered IgG glycosylation and the progression of HDC. A previous study reported that the polymorphisms at six loci (i.e., *FUT8*, *FUT6*/*FUT3*, *HNF1A*, *MGAT5*, *B3GAT1*, and *SLC9A9*) affect plasma levels of N-glycans [49], which could be used as the instrumental variables for future Mendelian randomization (MR) studies investigating the causal relationship between the altered IgG glycosylation and HDC. Fourthly, the information on medical treatment of the patients with HTN and/or T2DM was not completely collected. Consequently, the potential impact of medical treatment for HTN and/or T2DM on the associations between HDC and IgG glycans was unable to be addressed in this current study. Lastly, only the five-fold cross-validation of the model is performed, external validation of model performance is still needed in further studies.

In the current study, the IgG N-glycans were analyzed by HILIC-UPLC method, which is a profiling method for total IgG N-glycosylation. The detailed differences between the glycosylation profiles of the individual IgG subclasses associated with HDC remain to be investigated, which would be performed in our coming study. Future prospective studies with larger samples in multiracial populations and/or MR studies examining causal inference are warranted to validate the potential of IgG N-glycosylation biomarkers for personalized monitoring and prevention of HDC. Clinical exploitation of IgG N-glycan biomarkers will be facilitated by the existing broad application in clinical laboratories of UPLC. This study shows the potential clinical application of N-glycans as dynamic biomarkers (e.g., of routine diagnostic and prophylactic test for HDC patients). Future longitudinal and prospective studies would provide a better understanding of the transition from HTN or T2DM to HDC and allow us to generate glycan-based markers for use in earlier detection of the risk of progression towards HDC. As such, healthy lifestyle intervention and therapeutic decision-making could be delivered timely for the HDC prophylaxis from the perspectives of predictive, preventive and personalized/precision medicine.

## 5. Conclusions

This study presents conclusive examples of population-specific N-glycosylation of IgG associated with HDC in three northwestern Chinese Muslim ethnic minorities the KGZ, TJK, and UIG, as well as a Han Chinese population, respectively. These findings offer an opportunity to understand how IgG N-glycosylation profiles may be developed as potential biomarkers indicative of pro- or anti-inflammatory states shared by HTN, T2DM, and HDC. Further prospective follow-up studies exploring the onset of HDC using glycomics are expected to provide a more comprehensive understanding of specific glycomic biomarkers that may serve as tools for personalized monitoring or to help prevent the progression from HTN or T2DM towards HDC.

## Figures and Tables

**Table 1:** Demographic and biochemical characteristics of the study subjects in the pooled samples from Chinese Muslim ethnic minorities and the Han Chinese samples.

	Pooling of Chinese Muslim Ethnic Minorities					Han Chinese				
Variables	HDC	HTN	T2DM	Controls	*p* *	HDC	HTN	T2DM	Controls	*p* *
	(*n* = 67)	(*n* = 183)	(*n* = 51)	(*n* = 183)		(*n* = 72)	(*n* = 112)	(*n* = 50)	(*n* = 165)	
Gender (male%)	37 (55.22%)	71 (38.80%)	16 (31.37%)	76 (41.53%)	4.80 × 10^−2^	37 (51.39%)	58 (50.43%)	23 (46.00%)	26 (15.76%) ^#^	<1.00 × 10^−3^
Age (years)	63 (54, 69)	59 (48, 67)	54 (43, 68)	51 (40, 61) ^$,#^	<1.00 × 10^−3^	66 (49, 76)	49 (43, 54)	54 (46, 73)	46 (42, 50) ^&,#^	<1.00 × 10^−3^
BMI (kg/m^2^)	26.23 ± 4.52	27.04 ± 4.73	26.27 ± 4.39	25.85 ± 4.69	1.06 × 10^−1^	24.97 (23.03, 27.04)	25.68 (23.84, 27.76)	24.22 (21.73, 27.53)	23.11 (21.57, 25.08) ^#^	<1.00 × 10^−3^
SBP (mmHg)	151.03 ± 21.47	147.13 ± 21.72	116.27 ± 9.69	115.35 ± 10.62 ^$,#^	<1.00 × 10^−3^	148.88 ± 15.77	132.77 ± 10.53	116.00 ± 16.16	104.87 ± 7.38 ^$,#^	<1.00 × 10^−3^
DBP (mmHg)	93.78 ± 13.42	91.82 ± 14.06	72.84 ± 8.10	73.02 ± 8.93 ^$,#^	<1.00 × 10^−3^	83.67 ± 10.31	93.13 ± 6.01	71.92 ± 10.14	68.57 ± 3.84 ^&,$,#^	<1.00 × 10^−3^
FBG (mmol/L)	7.40 (6.70, 8.10)	5.30 (5.00, 5.80)	7.00 (6.70, 7.80)	5.20 (4.90, 5.80) ^&,#^	<1.00 × 10^−3^	7.37 (5.60, 8.49)	5.32 (5.07, 5.78)	7.58 (6.77, 7.87)	5.03 (4.76, 5.36) ^&,#^	<1.00 × 10^−3^
TC (mmol/L)	4.71 (3.75, 5.34)	4.71 (4.06, 5.46)	4.58 (3.94, 5.19)	4.76 (4.18, 5.61)	3.97 × 10^−1^	4.59 ± 1.47	5.19 ± 0.93	4.59 ± 1.15	5.06 ± 0.92 ^&,#^	7.00 × 10^−3^
TG (mmol/L)	3.10 (2.47, 3.70)	3.07 (2.44, 3.70)	3.10 (2.76, 3.68)	3.19 (2.65, 3.78)	3.48 × 10^−1^	1.45 (1.05, 2.17)	1.52 (1.03, 2.38)	1.10 (0.71, 1.97)	0.98 (0.77, 1.44) ^$,#^	<1.00 × 10^−3^
HDL (mmol/L)	1.83 (1.51, 2.10)	1.64 (1.29, 2.04)	1.71 (1.27, 1.98)	1.78 (1.32, 2.13)	3.06 × 10^−1^	1.20 (0.95, 1.50)	1.49 (1.25, 1.70)	1.41 (1.07, 1.66)	1.66 (1.44, 1.89) ^&,#^	<1.00 × 10^−3^
LDL (mmol/L)	2.60 (1.67, 3.47)	2.29 (1.48, 3.17)	2.50 (1.80, 3.43)	2.17 (1.37, 3.28)	1.04 × 10^−1^	2.39 (1.86, 3.02)	2.76 (2.37, 3.26)	2.35 (1.80, 2.96)	2.65 (2.24, 3.19) ^&,#^	<1.00 × 10^−3^
Dyslipidemia (%)	57 (85.07%)	149 (81.42%)	47 (92.16%)	161 (87.98%)	1.57 × 10^−1^	35 (48.61%)	36 (32.14%)	17 (34.00%)	31 (18.79%) ^#^	<1.00 × 10^−3^

Data are shown as mean ± standard deviation or median (P_25_, P_75_); if the continuous variables were normally distributed, the *t*-test was used to analyze the differences between the groups. Otherwise, the Kruskal–Wallis test was used. χ2 test: Gender, dyslipidemia; BMI, body mass index; DBP, diastolic blood pressure; FBG, fasting blood glucose; HDC, hypertension and type 2 diabetes mellitus comorbidity; HDL, high-density lipoprotein cholesterol; HTN, hypertension; LDL, low-density lipoprotein cholesterol; SBP, systolic blood pressure; T2DM, type 2 diabetes mellitus; TC, total cholesterol; TG, total triglycerides; P_25_, the 25th percentile; P_75_, the 75th percentile; * *p* < 0.05 was considered statistical significance; ^&^ *p* < 0.0083 was considered statistical significance between HDC and HTN; ^$^ *p* < 0.0083 was considered statistical significance between HDC and T2DM; ^#^ *p* < 0.0083 was considered statistical significance between HDC and Controls.

**Table 2:** The discriminative performance of models for HDC in the pooled samples from Chinese Muslim ethnic minorities and the Han Chinese samples.

	Type of Model	Pooling of Chinese Muslim Ethnic Minorities				Han Chinese			
		Included Variables of Model	AUC (95%CI)	*p* *	Prediction Error	Included Variables of Model	AUC (95%CI)	*p* *	Prediction Error
HDC vs. HTN	baseline model	Age, gender, ethnicity, BMI, and dyslipidemia	0.653 (0.578, 0.728)	2.15 × 10^−4^	0.280 ± 0.010	Age, gender, BMI, and dyslipidemia	0.764 (0.686, 0.842)	1.49 × 10^−9^	0.249 ± 0.003
	glycan-based model	GP5, GP6, GP16, DG24, and DG54	0.678 (0.607, 0.748)	1.70 × 10^−5^	0.279 ± 0.001	GP20, DG2, and DG53	0.715 (0.637, 0.794)	8.38 × 10^−7^	0.281 ± 0.005
	combined model	Age, gender, ethnicity, BMI, dyslipidemia, GP5, GP6, GP16, DG24, and DG54	0.717 (0.651, 0.782)	1.52 × 10^−7^	0.291 ± 0.003	Age, gender, BMI, dyslipidemia, GP20, DG2, and DG53	0.828 (0.761, 0.896)	5.91 × 10^−14^	0.234 ± 0.008
HDC vs. T2DM	baseline model	Age, gender, ethnicity, BMI, and dyslipidemia	0.682 (0.584, 0.779)	6.60 × 10^−5^	0.439 ± 0.004	Age, gender, BMI, and dyslipidemia	0.609 (0.508, 0.710)	4.13 × 10^−2^	0.452 ± 0.001
	glycan-based model	GP5, GP16, and GP18	0.715 (0.622, 0.808)	7.35 × 10^−4^	0.420 ± 0.002	GP20 and GP24	0.663 (0.567, 0.759)	2.23 × 10^−3^	0.442 ± 0.009
	combined model	Age, gender, ethnicity, BMI, dyslipidemia, GP5, GP16, and GP18	0.747 (0.656, 0.838)	5.00 × 10^−6^	0.375 ± 0.010	Age, gender, BMI, dyslipidemia, GP20, and GP24	0.689 (0.594, 0.783)	4.00 × 10^−4^	0.426 ± 0.007
HDC vs. Controls	baseline model	Age, gender, ethnicity, BMI, and dyslipidemia	0.771 (0.709, 0.846)	1.70 × 10^−5^	0.251 ± 0.002	Age, gender, BMI, and dyslipidemia	0.871 (0.812, 0.931)	9.78 × 10^−20^	0.143 ± 0.001
	glycan-based model	GP5 and GP6	0.678 (0.606, 0.749)	5.64 × 10^−11^	0.269 ± 0.002	GP20	0.756 (0.685, 0.826)	4.01 × 10^−10^	0.203 ± 0.001
	combined model	Age, gender, ethnicity, BMI, dyslipidemia, GP5, and GP6	0.786 (0.727, 0.846)	4.03 × 10^−12^	0.238 ± 0.010	Age, gender, BMI, dyslipidemia, and GP20	0.901 (0.850, 0.951)	1.04 × 10^−22^	0.138 ± 0.001

AUC, area under the curve; BMI, body mass index; CI, confidence interval; DG, derived glycan; GP, glycan peak; HDC, hypertension and type 2 diabetes mellitus comorbidity; HTN, hypertension; SD, standard deviation; T2DM, type 2 diabetes mellitus; * *p* < 0.05 was considered statistical significance; The prediction error of five-fold cross-validation procedure was presented in the form of mean ± SD.

## Data Availability

The data presented in this study are available on request from the corresponding author. The data are not publicly available due to privacy restrictions.

## Supplementary Materials

The following are available online at https://www.mdpi.com/article/10.3390/jpm11070614/s1, Supplementary Table S1: The structures of 24 directly measured IgG glycan peaks by UPLC, Supplementary Table S2: Demographic and biochemical characteristics of the study subjects in the four Chinese ethnic groups: Kirgiz, Tajik, Uygur, and Han, Supplementary Table S3: The description of IgG glycome composition and the levels of IgG glycan traits in the HDC, HTN, T2DM patients, and healthy controls for the pooled samples from Chinese Muslim ethnic minorities and the Han Chinese samples, Supplementary Table S4: The associations of IgG N-glycan traits with HDC vs. HTN/T2DM/healthy individuals for the pooled samples from Chinese Muslim ethnic minorities and the Han Chinese samples, Supplementary Table S5: The differences of AUC between three type of models.

## Abbreviations

AUC	area under the receiver operating characteristic curve
BMI	body mass index
CI	confidence interval
CVD	cardiovascular disease
DBP	diastolic blood pressure
DG	derived glycan
FBG	fasting blood glucose
GP	glycan peak
HDC	hypertension and type 2 diabetes mellitus comorbidity
HDL	high-density lipoprotein cholesterol
HILIC	hydrophilic interaction liquid chromatography
HTN	hypertension
IgG	immunoglobulin G
LDL	low-density lipoprotein cholesterol
OR	odds ratio
ROC	receiver operating characteristic
SBP	systolic blood pressure
T2DM	type 2 diabetes mellitus
TC	total cholesterol
TG	triglycerides
UPLC	ultra-performance liquid chromatography
